# Survey of Potential Cancer Registry Items to Enhance the Usefulness of Cancer Registry Data

**DOI:** 10.31557/APJCP.2019.20.10.3173

**Published:** 2019

**Authors:** Yoo-Kyung Boo, Hyun-Sook Lim, Young-Joo Won

**Affiliations:** 1 *Department of Healthcare Administration, Dankook University, Cheonan-si, Chungnam,*; 2 *Department of Public Health Administration, Hanyang Women’s University, Seoul,*; 3 *Division of Cancer Registration and Surveillance, National Cancer Center, Goyang, Korea. *

**Keywords:** Cancer registration, data collection, variable, availability, Korea

## Abstract

**Background:**

Cancer registry data can help plan for cancer services and to identify where further progress is needed, in order to improve the lives of patients with cancer. This study investigated the possibility of collecting additional information and the priority of the information by examining other cancer registry items. We aimed to suggest additional data items to be collected to enhance the usefulness of cancer registry data.

**Methods:**

We examined items that could potentially be added by comparing the cancer registration items in five foreign registries and large hospitals in Korea. Based on the foreign and domestic hospital cancer registry data, a questionnaire survey was administered to 272 cancer registry workers nationwide and 10 cancer experts to investigate the possibility of expanding the variables. The proportion and rank of each item were analyzed.

**Results:**

There were similar items for demographic information and cancer diagnosis between foreign cancer registries and the Korea Central Cancer Registry (KCCR). However, the KCCR had fewer items for staging, treatment, and follow-up. There were 29 items to be collected with high priority. Items under demographic information included date of birth, race and country of birth. Items for cancer diagnosis included type of cancer, smoking history and type of pathologic test. Treatment information included the date of treatment, chemotherapy and radiation. Items under the stage and prognostic factors included TNM stage, collaborative stage, and comorbidities. Finally, items under follow-up information included survival, cancer state and recurrence information.

**Conclusions:**

Cancer registration workers and cancer experts generally agreed on the need to expand the essential items for cancer registration. The findings of this study will be useful for devising plans to expand cancer registration items.

## Introduction

Cancer registration is a method of systematically and continuously collecting cancer data, including demographic, diagnostic, treatment, and outcome information (Moore, 2013). Accurate, complete, and timely cancer registry data could serve as useful information for assessing cancer incidence, monitoring and evaluation of the national cancer control program, as well as cancer research (Bray and Parkin, 2009). 

After the Korea Central Cancer Registry (KCCR) project was launched in 1980, the Korea National Cancer Incidence Database was established in South Korea in 1999 and has served as a foundation for national cancer statistics, from the calculation of cancer incidence to the assessment of survival and prevalence. Internationally, quality of cancer incidence statistics of Korea have been recognized by their inclusion in the “Cancer Incidence in Five Continents” volumes IX (2007), X (2013), and XI (2017) (http://ci5.iarc.fr/Default.aspx) published by the International Agency for Research on Cancer.

The World Health Organization suggests national cancer monitoring systems to measure the national and local cancer burden, socioeconomic differences, costs, individual and biological factors, and health behaviors, but many of these items are difficult to identify or collect (Ryerson and Massetti, 2017). As such, the cancer registries of Korea also have shortcomings in the collection of information about treatment and its outcomes (Soon et al., 2013). Therefore, continuous efforts to expand the scope of registry and items are essential to addressing the limitations of the national cancer registry data and to increase their usefulness. To this end, the KCCR has added the stage variable according to the Surveillance Epidemiology and End Results (SEER) summary stage. Additionally, collaborative staging (CS) information for four cancers (stomach, colon, breast, and uterine cervix), the path of diagnosis, laterality, differentiation, and site of metastasis has also been collected since 2012. 

Multiple aspects must be considered to expand the cancer registration items. First, we must consider whether the information can be drawn from medical records and whether the relevant information could be collected for all cancers and from all hospitals. Moreover, accurate information should be collected via standardized guidelines, computer programs, and education for data abstractors (medical record administrators [MRAs]).

Thus, this study aimed to investigate the additional cancer registration items that could be collected by examining foreign cancer registries and large hospitals in Korea. Further, we assessed the need to expand items and the priority of the items in order to present potential cancer registry items to be added. 

## Materials and Methods

**Figure 1:**
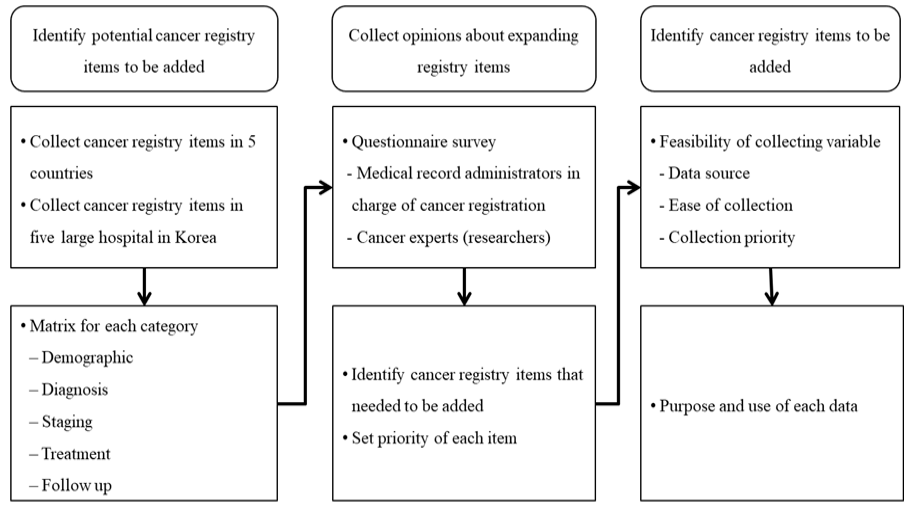
Study Flow Chart

This study was conducted in three stages (Figure 1). First, cancer registry items in five foreign registries and large hospitals in Korea were compared with those of the KCCR to identify items that could potentially be added to the KCCR database. Second, the identified items were surveyed among MRAs, who are the actual workers in charge of cancer registration in hospitals, to assess the need to collect these items and the priority of the items. Finally, the same items were presented to cancer experts to obtain information about essential items, the reason for selection, and the main areas of use. The methodology employed in this study was identical to that used in a study that investigated the need to collect data and factors hindering the utilization of the national program of cancer registries and clinical researchers in the United States (Zachary et al., 2015). 

*Identification of potential cancer registry items by comparing foreign and domestic data*

We compared the cancer registry items in five foreign cancer registries and five hospital cancer registries with reference to the KCCR items (Table 1). Cancer registry items collected by the North American Association of Central Cancer Registries (NAACCR), National Health Services (NHS) Cancer Registration Dataset, European Network of Cancer Registries (ENCR), Japanese Association of Cancer Registries (JACR) and Cancer Council Australia (CCA) were categorized into demographic information, cancer diagnosis, treatment, staging and prognostic factors, and follow-up for comparison. In addition to the cancer registry items submitted to the KCCR by five large hospitals in Korea, we investigated and compared additional items collected by these hospitals. 

*Identification of potential cancer registry items and analysis of their usefulness based on a consensus among cancer registration workers (MRAs) and cancer experts (researchers)*

We assessed the need to add items identified in foreign and domestic cancer registries to the KCCR, the priority of collection, the purpose of collection, and use of information in terms of developing a monitoring system, assessing cancer prevention and treatment effects, and utilizing data in cancer research. The survey was conducted among MRAs and cancer experts. 

The results were structured such that they could rate the need for each additional cancer registry item on a three-point scale (highly needed, needed, not needed) and the priority of the items by numbering them. In addition, MRAs were also asked to write about additional items that were not included in the survey. The results of the need for each item were presented as the total number of responses and percentage for each scale. The results of the priority of the items were presented as the rank of each item in the corresponding category and its mean and standard deviation. 

The results for cancer experts were structured such that they could rate the priority of each cancer registry item under each category and the purpose of collecting data for each item. Further, the cancer experts were asked to provide suggestions to enhance the usefulness of cancer registry data. The items and contents of the results were reviewed, modified, and validated by four investigators and participating researchers from the KCCR. The survey was conducted among 500 MRAs and 36 cancer researchers who were members of the subcommittee for the Third-Term Comprehensive Plan for Cancer Control.

**Table 1:** Items Collected in the Korea Central Cancer Registry

Category	Items
Demographic information	Hospital number, patient registration number, resident registration number, name, sex, age, foreign citizen, homeless patient, occupation, address, zip code
Cancer diagnosis	Date of initial diagnosis, path of diagnosis, primary site code, area of primary cancer, laterality, morphology code, grade, method of diagnosis, date of admission, date of discharge
Cancer treatment	Application of treatment, type of treatment (surgery, chemotherapy, radiation, immune, hormone)
Staging and prognostic factors	Staging (SEER code required, other staging information may be added), site of metastasis (enter site of metastasis at the time of diagnosis)
Follow-up*	Date of expiration, cause of death
Management information	Century of born, year of data, date of data entry, name of person who entered data, medical record administrator’s license No.

*, Enter if the patient was dead at the time of registration

**Table 2:** Comparison of Cancer Registry Items by Country (Excluding Management information)

Sources		Demographic information	Cancer diagnosis	Treatment	Staging and prognostic factors	Follow-up	Total
KCCR	Korea Central Cancer Registry (Korea)	11	9	2	2	2	26
NAACCR	North American Association of Central Cancer Registries (US)	83	48	97	248	80	556
NHS	National Health Services Cancer Registration Dataset (UK)	11	9	20	31	8	79
ENCR	European Network of Cancer Registries (EU)	6	19	6	18	3	52
JACR	Japanese Association of Cancer Registries (Japan)	6	8	9	1	1	25
CCA	Cancer Council Australia (Australia)	8	11		breast Selective	breast Selective	19

**Table 3:** Need for Potential CancerRegistry Items and Their Prioritization by Category (Survey among MRAs)

	Highly needed		Needed		Not needed		Missing		Priority		
	N	%	N	%	N	%	N	%	Rank	Mean	SD
Demographic information											
Date of birth	86	31.6	117	43.0	66	24.3	3	1.1	2	3.0	2.3
Place of birth	64	23.5	136	50.0	70	25.7	2	0.7	4	3.4	1.8
Country of birth	86	31.6	142	52.2	42	15.4	2	0.7	1	2.8	1.5
Type of residence (urban/rural)	76	27.9	163	59.9	32	11.8	1	0.4	3	3.2	1.6
Race	78	28.7	146	53.7	47	17.3	1	0.4	5	3.6	1.7
Ethnicity	54	19.9	127	46.7	90	33.1	1	0.4	6	5	1.7
Marital status at diagnosis	43	15.8	150	55.1	79	29	0	0.0	7	5.2	1.8
Cancer diagnosis											
Symptoms	139	51.1	116	42.6	15	5.5	2	0.7	2	3.3	2.5
Medical history	147	54.0	112	41.2	10	3.7	3	1.1	1	3.3	2.3
Smoking history	155	57.0	109	40.1	6	2.2	2	0.7	6	4.6	2.6
Date of multiple cancer diagnosis	140	51.5	108	39.7	21	7.7	3	1.1	4	4.1	2.1
Concurrent cancer	153	56.3	102	37.5	15	5.5	2	0.7	5	4.3	2.1
Type of cancer	166	61.0	90	33.1	14	5.1	2	0.7	3	3.5	2
Date of specimen submission	79	29.0	122	44.9	64	23.5	7	2.6	8	7.1	2.1
Type of specimen	79	29.0	118	43.4	68	25	7	2.6	9	7.5	1.8
Type of pathologic test	96	35.3	131	48.2	41	15.1	4	1.5	7	6.8	2.3
Name of pathologist	33	12.1	98	36.0	134	49.3	7	2.6	10	9.5	1.8
Pathology laboratory	42	15.4	121	44.5	103	37.9	6	2.2	11	9.8	1.8
Cancer treatment											
Date of treatment	131	48.2	110	40.4	29	10.7	2	0.7	1	2.6	2.2
Treatment code	121	44.5	112	41.2	35	12.9	4	1.5	2	3.4	2.2
Radiation therapy	116	42.6	112	41.2	39	14.3	5	1.8	4	3.6	1.7
Chemotherapy	121	44.5	118	43.4	27	9.9	6	2.2	3	3.6	1.8
Palliative treatment	101	37.1	131	48.2	35	12.9	5	1.8	6	5.1	2.2
Reason for non-treatment	74	27.2	140	51.5	53	19.5	5	1.8	7	6.1	2.2
Treatment outcomes	138	50.7	108	39.7	21	7.7	4	1.5	5	4.1	2.2
30-day readmission	70	25.7	133	48.9	65	23.9	4	1.5	8	6.8	2
Follow-up care	74	27.2	153	56.3	41	15.1	4	1.5	9	7.1	2.1
Staging and prognostic factors											
Clinical TNM stage	139	51.1	108	39.7	18	6.6	7	2.6	2	2.6	2.2
Pathologic TNM stage	169	62.1	89	32.7	8	2.9	6	2.2	1	1.9	1.5
TNM version	108	39.7	125	46.0	30	11	9	3.3	3	4.2	2.3
Pediatric cancer staging system	108	39.7	117	43.0	38	14	9	3.3	7	5.8	2.4
Pre-treatment CS	115	42.3	131	48.2	20	7.4	6	2.2	4	4.2	1.6
Post-treatment CS	117	43.0	124	45.6	25	9.2	6	2.2	5	5.0	1.6
CS-site of metastasis	128	47.1	119	43.8	20	7.4	5	1.8	6	5.2	1.7
CS-site-specific factor 1-25	91	33.5	127	46.7	46	16.9	8	2.9	8	7.0	1.6
Comorbidity and complication 1-10	85	31.3	136	50.0	44	16.2	7	2.6	9	7.5	1.9
Follow-up information											
Date of final contact	67	24.6	128	47.1	67	24.6	10	3.7	4	4.9	3.2
Age at final contact	60	22.1	125	46.0	77	28.3	10	3.7	6	6.0	3.4
Facility of final contact	52	19.1	134	49.3	78	28.7	8	2.9	9	6.9	3.3
Survival	139	51.1	112	41.2	15	5.5	6	2.2	1	2.8	2.4
Duration of survival	139	51.1	114	41.9	13	4.8	6	2.2	2	3.5	2.6
Cancer state	108	39.7	119	43.8	34	12.5	11	4	3	4.6	2.9
Activities of daily living	54	19.9	145	53.3	64	23.5	9	3.3	8	6.7	2.9
Follow-up information											
Source of follow-up	49	18.0	131	48.2	82	30.1	10	3.7	10	8.0	2.9
Address at follow-up	41	15.1	126	46.3	94	34.6	11	4.0	11	9.3	3.0
Date of initial recurrence	124	45.6	121	44.5	18	6.6	9	3.3	5	5.4	3.0
Type of recurrence	121	44.5	114	41.9	29	10.7	8	2.9	7	6.2	3.1
Place of death	57	21.0	116	42.6	91	33.5	8	2.9	12	10.2	3.1
Autopsy	41	15.1	131	48.2	88	32.4	12	4.4	13	10.4	3.1
Physician in charge	29	10.7	110	40.4	120	44.1	13	4.8	14	12.5	2.5

**Table 4:** Potential Cancer Registry Items Identified by Cancer Experts

Category	Required items	Major purpose	Main use
Demographic information	Date of birth	Personal identification	Alternative to resident registration number
	Race	Foreigner identification	Research on cancer trends in foreigners
	Country of birth	Foreigner identification	Research on cancer trends in foreigners
	Place of birth	Identification of geographical factors	Research on geographical/environmental associations with cancer
	Marital status at diagnosis	Identification of cause of cancer	Incidence and survival of particular cancers vary according to marital status
Cancer diagnosis	Type of Cancer (initial, recurrent)	Classification of cancer	Computation of recurrence rate
	Smoking history	Cause of specific cancer	Identification of its association with specific cancer
	Date of multiple primary cancer diagnosis	Confirmation of multiple primary cancer	Multiple primary cancer has poor prognosis and requires a wider range of treatment
	Concurrent cancer	Confirmation of unilateral organ	Identification of cancer in unilateral organ
	Medical history	Individual and family history related to cancer	Identification of factors related to cancer
	Symptoms	Symptoms related to specific cancer or asymptomatic	Identification of factors related to cancer
	Type of pathologic test	Confirmation of reliability of diagnosis	Confirmation of reliability of diagnosis
Cancer treatment	Date of treatment	Collect detailed treatment information	Identify treatment efficacy
	Chemotherapy agent	Collect detailed treatment information	Identify treatment efficacy
	Radiation therapy (type, dose, site)	Collect detailed treatment information	Identify treatment efficacy
	Treatment code	Collect detailed treatment information	Identify treatment efficacy
	Palliative treatment	Collect information about palliative treatment	Identify efficacy of palliative treatment
	Treatment outcomes (e.g., surgery)	Collect information about post-treatment state	Identify treatment efficacy
	Reason for non-treatment	Policy-related management, such as treatment support	Identify treatment efficacy
Staging and prognostic factors	TNM stage (clinical, pathologic)	Stage data	Determine staging and assess treatment efficacy
	CS (pre-treatment, post-treatment, site of metastasis)	Stage data	Determine staging and assess treatment efficacy
	CS- site-specific factor	Stage data	Determine staging and assess treatment efficacy
	Comorbidity and complication	Stage data	Determine staging and assess patient survival and outcomes
Follow-up information	Survival	Check for cancer progression	Compute survival rate
	Cancer state	Check for residual cancer	Compute disease-free survival rate
	Type of recurrence	Check for cancer progression	Compute recurrence rate
	Duration of survival	Check for cancer progression	Compute survival rate
	Date of initial recurrence	Check for cancer progression	Compute disease-free survival rate and recurrence rate
	Date of final contact	Check for cancer progression	Use in follow-up investigation

**Figure 2:**
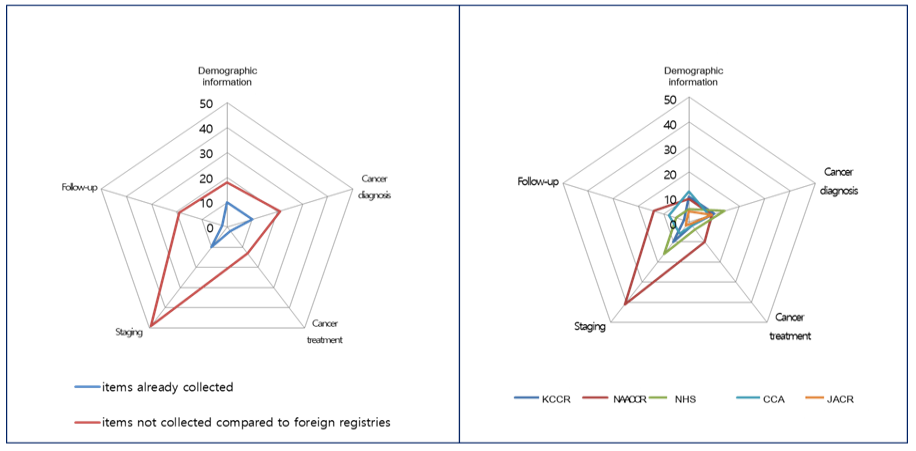
Comparison of the Distribution of Cancer Registry Items by Category

*Ethics statement*

This study utilized the outcomes of a project conducted as a part of the 2015 National Cancer Registry and Statistics Project by the National Health Promotion Fund. The questionnaire used in the study does not include personal information or any other content that required a consent form.

## Results

*Identification of potential cancer registry items by comparing foreign and domestic data*

After excluding “management information,” 26, 556, 79, and 52 items were collected from the KCCR, the NAACCR (2013), the NHS, and the ENCR (2015), respectively. The registries in the US and UK, and the ENCR collected more information about treatment, staging and prognostic factors, and follow-up than did KCCR. However, the JACR and CCA collected information regarding fewer items than did KCCR (Table 2, Figure 2). 

With reference to the items in the KCCR, the following foreign and Korean cancer registry items were collected in five categories (total number of items not shown) (Supplementary Tables). Under the category of “demographic information,” foreign cancer registries collected information on the place of birth, race or ethnicity, and type of residence (urban/rural). Some hospitals also collected information regarding marital status, telephone number, weight, and height. Under the category of “cancer diagnosis,” the KCCR and foreign cancer registries collected information about similar items in general. Some foreign and Korean hospital registries additionally collected information about symptoms, medical history, diagnostic date of multiple primary cancer, pathologic results, tumor marker, drinking history, cancer size, and family history. 

Under the category of “cancer treatment,” the KCCR, foreign cancer registries, and Korean hospital registries all collected information about surgery, chemotherapy, radiation therapy, immune therapy, and hormone therapy. Some foreign and Korean hospital registries additionally collected information about the date of treatment and treatment outcomes; particularly, the NAACCR collected information about re-admission within 30 days. 

Under the category of “staging and prognostic factors,” cancer registries commonly collected information about staging (SEER, Extent of Disease) and Tumor, Node, and Metastasis (TNM) stage. Information about the pediatric stage was only collected by the NAACCR, while information about CS was collected by the NAACCR and Korean registries. At one Korean hospital, information about toxic substances, ionizing radiation, exposure to second-hand smoke and exposure to asbestos were also collected. 

Under the category of “follow-up,” the KCCR only collected information regarding the date of death and cause of death, while foreign cancer registries collected more detailed information, including that regarding vital status, survival period, type of recurrence, and physicians. Korean hospital registries also collected additional information on follow-up items other than the date and cause of death.

*Selection of additional cancer registry items based on consensus among cancer registration workers*

Table 3 shows the results of the need and priority of additional cancer registry items reported by 272 cancer registration workers out of 500 participants (54.4%).

Under the category of “demographic information,” items regarding date of birth (n=86, 31.6%), country of birth (n=86, 31.6%), and race (n=78, 28.7%) were reported to be “highly needed.” Put in order of priority they were country of birth, date of birth, type of residence (urban/rural), place of birth, race, ethnicity, and marital status at diagnosis. 

Under the category of “cancer diagnosis,” more than 50% of the participants reported that information regarding the type of cancer, smoking history, concurrent cancer, medical history, date of multiple cancer diagnosis, and symptoms was highly needed. Placed in order of priority they included medical history, symptoms, type of cancer, date of multiple cancer diagnosis, concurrent cancer, smoking history, type of pathologic tests. 

Under the category of “cancer treatment,” more than 40% of the participants reported that information regarding treatment outcomes, date of treatment, treatment code, chemotherapy, radiation therapy, and palliative treatment were highly needed. Put in order of priority, they were the date of treatment, treatment code, chemotherapy, radiation therapy, treatment outcomes, palliative treatment, reason for non-treatment.

Under the category of “staging and prognostic factors,” more than 50% of the participants reported that information regarding pathologic TNM stage and clinical TNM stage were highly needed. Put in order of priority they were pathologic TNM stage, clinical TNM stage, TNM version used in the cancer registry, pre-treatment CS, post-treatment CS, CS-site of metastasis, and pediatric cancer staging. 

Under the category of “follow-up information,” more than 50% of the participants reported that information regarding survival (n=139, 51.1%) and duration of survival (n=139, 51.1%) were highly needed. Put in order of priority, they were survival, duration of survival, cancer state, date of final contact, date of initial recurrence, age at final contact, and type of recurrence.

*Selection of additional cancer registry items and use of information based on consensus among cancer experts*

Table 4 shows the results reported by 10 cancer experts out of 36 participants. Under the category of “demographic information,” the experts reported that information regarding date of birth was the most needed, followed by race, country of birth, place of birth, and marital status at diagnosis. Particularly, the experts stated that information regarding date of birth is needed when there is a limitation with using resident registration numbers. The experts stated that information regarding race, country of birth, and place of birth needed to be collected for research on the cancer incidence trends among foreigners, as there is an increasing foreign population in Korea. 

Under the category of “cancer diagnosis,” the experts reported that information regarding the type of cancer (initial, recurrent) was the most important, followed by those of smoking history, date of multiple primary cancer diagnosis, concurrent cancer, medical history, symptoms, and types of pathologic tests. Information regarding the date of multiple primary cancer diagnosis and type of cancer (initial, recurrent) were needed to classify multiple primary cancers. Information regarding types of pathologic tests was needed to confirm the accuracy of diagnosis and reliability of pathologic tests. 

Under the category of “cancer treatment,” the experts reported that information regarding date of treatment, the agent used for chemotherapy, radiation therapy, treatment code, palliative treatment, treatment outcomes, and reason for non-treatment need to be collected. Information regarding treatment codes and date of treatment are important for evaluating treatment outcomes; for radiation therapy, information about completion of treatment, as opposed to total treatment dose and area, is needed. Further, the experts stated that information regarding chemotherapy is needed to evaluate the efficacy of chemotherapy agents. Some experts stated that it would be possible to estimate the percentage of cancer patients participating in a clinical trial for a novel drug if relevant information was collected. 

Under the category of “staging and prognostic factors,” the experts reported that information regarding TNM stage, CS, CS-site specific factor, comorbidity, and complications need to be collected. The experts agreed that these items are required to evaluate the efficacy of treatment.

Under the category of “follow-up information,” the experts reported that information regarding survival, cancer state, type of recurrence, duration of survival, date of initial recurrence, and date of final contact need to be collected. Information regarding the date of final contact is needed to verify the accuracy of the follow-up data, and those of survival and duration of survival are needed to calculate the survival rate. Information regarding the date of initial recurrence is needed to compute the disease-free survival rate, and that regarding the type of recurrence is needed to determine whether the recurrence is local or remote and whether metastasis occurred. 

## Discussion

This study suggested additional data items to be collected to enhance the usefulness of KCCR data, based on foreign cancer registries and the specified large hospitals in Korea. A comparison of foreign cancer registry items and the KCCR registry items revealed that they collected information regarding similar items for “demographic information” and “cancer diagnosis,” but the KCCR collected information regarding fewer items under the categories of “treatment,” “staging and prognostic factors,” and “follow-up.” Under the category of “demographic information,” foreign cancer registries collected information such as place of birth, race, and type of residence (urban/rural). Under the category of “cancer diagnosis,” the KCCR and foreign cancer registries mostly collected information regarding similar items. In the NAACCR, information regarding pathologic results were emphasized for collection. Under the category of “staging and prognostic factors,” the KCCR and foreign cancer registries collected information about the summary stage and TNM stage. Information about pediatric staging was collected only in the NAACCR, while that regarding CS was only collected in the NAACCR and Korean registry. Under the category of “follow-up,” the KCCR only collected information regarding date of death and cause of death, while the foreign cancer registries collected information about recurrence, survival, address at follow-up, and physician in charge. In Australia, cancer registries in each state differ by the inclusion of some additional items depending on the characteristics of the state. Korea could follow this example, by maintaining common items and adding additional items relevant to the region and type of cancer to establish a differentiated database. 

The NAACCR institutes cancer registry certification for each state, which could also be applied in Korea. A program to certify data collection and management in hospital cancer registries could be developed as a measure to enhance the quality of the data. Hospital cancer registries collect data either directly from patients’ medical records or from an established database based on a system that analyzes discharged patients. 

In this study, we investigated additional items, information of which could be collected by the KCCR, because hospital registries would collect more information than required by the KCCR for research purposes. Under the category of “demographic information,” hospital registries collected information regarding marital status, telephone number, weight, and height at diagnosis. Under the category of “cancer diagnosis,” information regarding symptoms, smoking history, diagnostic date of multiple cancers, tumor marker, and cancer size and family history were collected. Under the category of “cancer treatment,” information about the date of treatment, palliative care at this facility, detailed radiation therapy data, and treatment outcomes were collected. Under the category of “staging and prognostic factors,” information regarding TNM and other staging information and comorbidity/complication data were collected. Under the category of “follow-up,” more detailed information, such as those of cause of death, survival period, and cancer status (complete remission, partial remission) were collected with more active follow-up. As shown here, hospital cancer registries are collecting and utilizing more detailed data than the KCCR, so it would be less challenging for the KCCR to expand the number of registry items to be collected in the future. Further, the inclusion of additional cancer registry items by linking with the hospital database could improve the quality and usefulness of the KCCR data. However, additional studies are needed to measure the increased work burden imposed upon cancer registration workers, which could limit the expansion of cancer registry items, and to investigate possible solutions to this problem. 

In the present study, MRAs and cancer experts mostly agreed on the registry items that should be added, and there was a consensus among the two groups regarding the need to add more items to the national cancer registration dataset. In the survey of the need for additional items, which are registered in foreign cancer registries but not in the KCCR, and priority of registration, hospital MRAs and cancer experts reported that information regarding date of birth, country of birth, and race are highly needed items under the category of “demographic information.” Considering the current environment with an increased entry of foreigners into the country, these additional items could be used as sources of data for relevant policymaking and research. Under the category of “cancer diagnosis,” the experts agreed that information regarding type of cancer, medical history, and smoking history should be collected. This finding corroborates an American study that investigated the need for additional cancer registry items and confirmed the need to collect additional smoking-related information (Zachary et al., 2015). Under the category of “cancer treatment,” the experts reported the need to collect data regarding the date of treatment, chemotherapy agent, and radiation therapy. These items could be collected from the electronic medical record systems and medical record management systems of hospitals. Thus, the standard for each item and collection procedure could be suggested based on a discussion among hospital personnel, KCCR, and researchers. Under the category of “staging,” the experts reported the need to collect information regarding TNM stage, CS, and comorbidity/complication. It is beneficial to collect information regarding both TNM and CS in terms of potentially complementing each other when either is omitted. Moreover, the experts suggested the need to improve the current collection of staging information. Although information regarding comorbidity can be collected from medical record management systems, its scope is too wide and vague; thus, data regarding comorbidity should be collected based on a clear criterion or a link to another source. Under the category of “follow-up,” the experts reported the need to add information regarding survival, cancer state, type of recurrence, and duration of survival. The experts determined that linking the cancer registry data with other data sources, such as big healthcare data, would enable an indirect collection of follow-up data. Furthermore, it is important to improve the entry of cancer-related information into medical records to enhance the accuracy of cancer registry data, calling for the implementation of continuous and active quality improvement activities. More detailed information regarding diagnosis, staging and prognostic factors, treatment, and follow-up must be collected to expand cancer research using cancer registry data. However, a further subdivision of these categories may limit the collection and completeness of data. Thus, it is important to collect key data and add additional items for cancer research and monitoring. 

National cancer registries enable researchers to not only produce cancer statistics and identify time-series trends, but also to identify the causes of cancer and assess treatment effects by collecting demographic, socioeconomic, and clinical data. It is also possible to partially expand the scope of cancer registry data by linking them to external data sources. Therefore, the expansion of cancer registry items could provide information for computing novel statistical indices and enable the production of more advanced cancer statistics in Korea. 
